# Lithium nephrotoxicity

**DOI:** 10.1186/s40345-015-0028-y

**Published:** 2015-06-05

**Authors:** Abed N. Azab, Alla Shnaider, Yamima Osher, Dana Wang, Yuly Bersudsky, R. H. Belmaker

**Affiliations:** School for Community Health Professions, Soroka University Medical Center, Beer-Sheva, Israel; Nephrology Department, Soroka University Medical Center, Beer-Sheva, Israel; Bipolar Disorders Clinic, Beer-Sheva Mental Health Center, Faculty of Health Sciences, Ben-Gurion University of the Negev, Beer-Sheva, Israel; Bipolar Disorders Clinic, Hadassah Medical Center, Hadassah University Hospital, Jerusalem, Israel

**Keywords:** Bipolar disorder, Creatinine, Kidney, Lithium, Nephrotoxicity, Suicide

## Abstract

Reports of toxic effects on the kidney of lithium treatment emerged very soon after lithium therapy was introduced. Lithium-induced nephrogenic diabetes insipidus is usually self-limiting or not clinically dangerous. Some reports of irreversible chronic kidney disease and renal failure were difficult to attribute to lithium treatment since chronic kidney disease and renal failure exist in the population at large. In recent years, large-scale epidemiological studies have convincingly shown that lithium treatment elevates the risk of chronic kidney disease and renal failure. Most patients do not experience renal side effects. The most common side effect of polyuria only weakly predicts increasing creatinine or reduced kidney function. Among those patients who do experience decrease in creatinine clearance, some may require continuation of lithium treatment even as their creatinine increases. Other patients may be able to switch to a different mood stabilizer medication, but kidney function may continue to deteriorate even after lithium cessation. Most, but not all, evidence today recommends using a lower lithium plasma level target for long-term maintenance and thereby reducing risks of severe nephrotoxicity.

## Review

Lithium is a useful mood stabilizer for maintenance treatment of bipolar disorder (Belmaker 2004; Gershon et al. 2009). It has established antimanic effect and prophylactic efficacy against recurrence of affective episodes (Belmaker 2004; Gershon et al. 2009). Another advantage of lithium is its proven ability to reduce suicide attempts and suicidal death among bipolar patients. In recent years, concern has developed about the risk of lithium therapy in rare cases leading to chronic renal failure and even necessitating kidney dialysis. In this paper, we discuss these concerns about lithium therapy even as we hope that lithium with its unique clinical benefits will be used more widely in psychiatry. Experience in psychiatry with the late recognition of the serious metabolic side effects of atypical antipsychotics leads us to believe that it is better to confront concerns about this serious side effect now, rather than allowing the issue to polarize into a situation where there are those clinicians “for lithium” and those clinicians “against lithium.”

Kidney function impairments in patients taking lithium were reported as early as the 1970s (McKnight et al. 2012; Markowitz et al. 2000; Kallner and Petterson 1995; Bucht and Wahlin 1980; Hestbech et al. 1977; Baylis and Heath 1978; Angrist et al. 1970). Lithium therapy has been associated with a number of renal function abnormalities.

### Nephrogenic diabetes insipidus

The most common renal side effect of lithium is of concentrating urine despite normal or elevated concentrations of the antidiuretic hormone vasopressin (Table 1). The concentrating defect leads to decreased urine osmolality and increased urine volume (polyuria). A urinary concentrating defect may occur without overt polyuria, but this is usually clinically insignificant because near-normal urinary volume rarely leads to the performance of urine osmolality tests.

**Table 1:** Controversies over lithium and the kidney (selected references)

	Yes	No
Lithium causes anatomic kidney damage	Markowitz et al. (2000)	Vestergaard et al. (1979)
Low-dose lithium prevents kidney damage	Aiff et al. (2014a)	Close et al. (2014)
Duration of lithium treatment predicts Li polyuria	Bendz et al. (2001)	Lepkifker et al. (2004)
Duration of lithium predicts reduced GFR	Bendz et al. (2010)	Smigan et al. (1984)

The incidence of nephrogenic diabetes insipidus (NDI) among lithium-treated patients varies greatly in different studies, with a prevalence range of 20 to 87 % (Baylis and Heath 1978; Vestergaard et al. 1979; Vestergaard and Amdisen 1981; Schou and Vestergaard 1988; Okusa and Crystal 1994; McKnight et al. 2012; Markowitz et al. 2000; Kallner and Petterson 1995; Bucht and Wahlin 1980; Boton et al. 1987). For example, in a comprehensive meta-analysis of studies comprising 1172 lithium-treated patients, Boton et al. (1987) reported a reduction in urinary concentrating ability in more than 54 % of patients while only 19 % had overt polyuria.

Many studies found that major factors affecting the incidence and severity of urinary concentrating defects among lithium-treated patients are as follows: duration of treatment (longer duration increases the risk of NDI), blood lithium level, and frequency of acute lithium intoxications (Vestergaard et al. 1979; Vestergaard and Amdisen 1981; Schou and Vestergaard 1988; Markowitz et al. 2000; Bucht and Wahlin 1980; Boton et al. 1987; Walker 1993; Turan et al. 2002; Timmer and Sands 1999; Bendz et al. 2001). On the other hand, Lepkifker et al. (2004) observed that the duration of lithium treatment and plasma concentrations were not associated with increased risk of urinary concentrating defect while episodes of lithium intoxication were more predictive and positively correlated. A urinary concentrating defect may occur as early as 2 to 4 months after the commencement of lithium (Boton et al. 1987; Smigan et al. 1984), but it becomes more evident after chronic treatment (Vestergaard and Amdisen 1981; Markowitz et al. 2000; Bendz et al. 2001; Phillips et al. 2008). NDI may develop even after cessation of lithium therapy (Paw et al. 2007). Additionally, several studies demonstrated that cessation of long-term lithium therapy does not always restore the urinary concentrating capacity of the kidney (Markowitz et al. 2000; Bendz et al. 2001; Khairallah et al. 2007) while others reported that it may alleviate the concentrating defect (Bucht and Wahlin 1980). These variations may be related to the stage of tubulointerstitial damage at which lithium was stopped. An early stage at which only *functional* tubulointerstitial damage has occurred may be fully reversible. On the other hand, a late stage at which irreversible *morphological* tubulointerstitial changes (fibrosis) have occurred will not be resolved by lithium cessation. Importantly, patients with polyuria that do not consume sufficient amounts of fluids are at a high risk of becoming volume-depleted, further increasing the risk of lithium toxicity (Vestergaard et al. 1979; Vestergaard and Amdisen 1981; Smigan et al. 1984). Furthermore, patients who were concurrently treated with lithium and neuroleptic drugs had significantly higher rates of urinary concentrating defects than patients who were treated only with lithium. Consistently, antipsychotics (only)-treated patients had a higher incidence of urinary concentrating defects than matched healthy subjects (Bucht and Wahlin 1980). These observations suggest that antipsychotic drugs may contribute to the development of urinary concentrating defects.

Acute lithium treatment reduces the antidiuretic effect of vasopressin (Singer et al. 1972). Similarly, chronic lithium treatment was shown to reduce the antidiuretic effect of vasopressin by several possible mechanisms. The most established pharmacological treatment for lithium-induced NDI is the potassium-sparing diuretic amiloride (Boton et al. 1987; Timmer and Sands 1999; Feuerstein et al. 1981; Bedford et al. 2008). However, amiloride is likely to be effective only when there is a mild to moderate urinary concentrating defect that is potentially reversible. Among the suggested mechanisms by which amiloride alleviates lithium-induced urinary concentrating defect are as follows: *i*) blocking of epithelial sodium channel ENaC, resulting in reduced absorption of lithium into collecting duct cells (Walker et al. 1982), and *ii*) restoring to normal the expression levels of AQP2 and AQP3 (Bedford et al. 2008).

### Chronic kidney disease

According to the American National Kidney Foundation (2002), chronic kidney disease (CKD) is defined as either kidney damage or GFR <60 ml/min/1.73 m^2^ for ≥3 months. Kidney damage is defined as pathologic abnormalities or markers of damage, including abnormalities in blood or urine tests or imaging tests. The staging of CKD according to GFR (ml/min/1.73 m^2^) is as follows: Stage 1—kidney damage with normal or increased GFR (GFR ≥90); Stage 2—kidney damage with a mildly decreased GFR (GFR = 60–89); Stage 3—moderate reduction in GFR (GFR = 30–59); Stage 4—severe reduction in GFR (GFR = 15–29); and Stage 5—kidney failure (GFR <15 or dialysis) (American National Kidney Foundation 2002). End-stage renal disease (ESRD) describes a situation in which patients need dialysis or transplantation, irrespective of their level of kidney function (American National Kidney Foundation 2002).

Early studies questioned the association between lithium and chronic impairment in glomerular function (Vestergaard et al. 1979; Vestergaard and Amdisen 1981; Boton et al. 1987; Walker 1993; Walker et al. 1982; Bendz et al. 1983, 1994; Coskunol et al. 1997; Paul et al. 2010). In 1979, Vestergaard et al. (1979) examined renal function among 237 patients who had been treated with lithium for 0.5–17 years (average 5 years). They determined GFR by examining 24-h creatinine clearance and serum creatinine. It was found that lithium treatment reduced GFR only slightly and that the risk of developing ESRD was low (Bucht and Wahlin 1980). Two years later, these authors published a follow-up report on 184 patients of the same population in which they re-examined renal function (Vestergaard and Amdisen 1981). For 37 patients, lithium had been discontinued while the other 147 were still on lithium. None of the patients in either group had a reduction in GFR (Vestergaard and Amdisen 1981). Impairments in urinary concentrating ability had however progressed in the lithium-treated group. Subsequently, Walker et al. (1982) reported that the results of renal biopsy samples analyzed for interstitial fibrosis showed no difference between lithium-treated patients and those who did not receive lithium. However, abnormal serum creatinine levels were significantly higher in lithium-treated patients as compared to those who did not receive lithium, suggesting an impaired GFR (Walker et al. 1982). A meta-analysis study by Boton et al. revealed that 15 % of 1172 patients on chronic lithium therapy displayed only mild reduction in GFR (Timmer and Sands 1999). Bendz et al. (1983) examined glomerular function in outpatients who received short-term and long-term lithium treatment. They found that 3 % had abnormal glomerular function while 51 % had impaired tubular function. Eleven years after the previous study (Bendz et al. 1983), they reported that among 142 patients who received lithium for more than 15 years, a reduction in GFR was observed in 21 % of the patients (Bendz et al. 1994). The results also revealed that co-administration of lithium with other psychotropic drugs or somatic medications increased the risk of altered renal function. Coskunol et al. (1997) compared 107 lithium-treated patients to 29 non-lithium-treated patients matched for age and sex. They found that plasma creatinine levels and creatinine clearance did not significantly differ between the two groups. In addition, they found that there was no significant association between creatinine clearance and the duration (or dosage) of lithium treatment (Coskunol et al. 1997). A meta-analysis of 23 studies conducted by Paul and co-workers concluded that: “any lithium-associated increase in serum creatinine is quantitatively small and of questionable clinical significance;” this is despite a significant increase in serum creatinine levels in several studies that were analyzed (Paul et al. 2010). A comprehensive meta-analysis of 385 studies by McKnight et al. (2012) found a non-significant (*p* = 0.15) reduction in GFR and a low risk for developing ESRD among lithium-treated patients.

Despite the uncertainty reported in the studies cited above, other studies have suggested that long-term lithium therapy increases the risk of CKD (Markowitz et al. 2000; Turan et al. 2002; Bendz et al. 2001, 2010; Aiff et al. 2014a; Kripalani et al. 2009; McCann et al. 2008; Presne et al. 2003). One of the studies that demonstrated deleterious and mostly irreversible renal damage after chronic lithium therapy was that by Markowitz et al. (2000), which included 24 patients (average lithium treatment duration 13.6 years) with renal insufficiency. Renal biopsy results revealed a chronic tubulointerstitial nephropathy in all patients, with associated cortical and medullary tubular cysts and dilation in 62.5 and 33.3 % of patients, respectively (Markowitz et al. 2000). Nine of 19 patients progressed to ESRD despite lithium withdrawal (eight of nine had an initial serum creatinine above 2.5 mg/dL and one of ten had an initial serum creatinine below 2.5 mg/dL). On the other hand, three patients with initial creatinine levels below 2.1 mg/dL had subsequent improvement in renal function (Markowitz et al. 2000). The authors concluded that serum creatinine levels higher than 2.5 mg/dL are the only significant predictor of progressing to ESRD.

The Markowitz et al. (2000) study was retrospective, biopsy-based from a selected renal biopsy database. Of 6514 biopsies that made up the database, only 24 (0.37 %) were those of lithium-treated patients. Moreover, it is not clear whether lithium therapy was indeed the sole reason for kidney disease and referral to renal biopsy. One of the patients who discontinued lithium subsequently committed suicide, underscoring the complexity of discontinuing lithium even in the presence of kidney disease. A study by Presne et al. (2003) also demonstrated the correlation between long-term lithium therapy and CKD. It included 54 patients for whom chronic lithium treatment was apparently the only cause of renal disease (during the study period, six other patients were excluded because they had evidence of other causes for renal disease). Another 20 patients on chronic lithium therapy were referred for systematic renal biopsy. Among those 74 patients (mean age at onset of lithium therapy 42.9 years, mean duration of lithium therapy 19.8 years), 12 (16%) reached ESRD at a mean age of 65 years (Presne et al. 2003). Renal function was determined by measuring serum creatinine and creatinine clearance. Lithium-induced chronic nephropathy seemed to be a slowly progressing disease. The average latency between initiation of lithium and the progression to ESRD was estimated at 20 years (Presne et al. 2003). The severity of interstitial fibrosis on renal biopsy was associated with treatment duration and cumulative dose of lithium.

A survey of lithium-induced ESRD in French dialysis centers was conducted. Among 10,726 patients surveyed, 24 lithium-treated patients (0.22 %) had ESRD (Presne et al. 2003). The authors found that the duration of lithium treatment is the most significant factor that negatively affects renal function. A cohort study of kidney damage in long-term lithium patients demonstrated a significant correlation between treatment duration and the frequency of decreased GFR and urinary concentrating ability (Bendz et al. 2001). Eighty-six patients were on lithium (mean age 58, mean treatment duration 16 years) and 42 patients (mean age 57, mean treatment duration 10 years) were off lithium at follow-up time. For lithium-treated patients, the frequency of reduced urinary concentrating ability and increased serum creatinine significantly increased during mean follow-up periods of 8 and 10 years, respectively (Bendz et al. 2001). The authors concluded that among patients on long-term lithium therapy, the prevalence of significant nephropathy increases with time and that reduced GFR is less common than a reduction in urinary concentrating ability (Bendz et al. 2001). Similarly, a study by McCann et al. (2008) examined 38 patients who had been treated with lithium for a mean of 6.9 years (short-term) and 21 patients who were on lithium for a mean of 14.2 years (long-term). They found that long-term treatment patients had a significantly higher serum creatinine and urea levels (McCann et al. 2008). However, this study had several limitations including the relatively small sample size and being a retrospective, cross-sectional study. These and other limitations led the authors to emphasize that although “regression analysis does show that longer duration of lithium use is associated with higher creatinine level,…, this is not necessarily associated with a clinically relevant abnormalities in renal function” (McCann et al. 2008).

Bendz et al. (2010) assessed the prevalence of ESRD that required renal replacement therapy (RRT) in a lithium-treated population in Sweden. They found that 2202 subjects were on RRT among the surveyed general population (2,684,157), a prevalence of 0.8/1000. On the other hand, among a population of 3369 lithium-treated patients, 18 were on RRT (5.3/1000), representing a sixfold increase in RRT prevalence (Bendz et al. 2010). For the latter 18 patients on RRT, lithium treatment was apparently the only reason for ESRD (lithium-treated patients on RTT were excluded if they had other reasons that may have caused ESRD). The average age at the start of RRT among those 18 patients was 62 years (range 46–74), and the average duration of lithium treatment before initiation of RTT was 23 years (range 12–35). Moreover, of the 3369 lithium-treated patients, 41 had plasma creatinine levels above 150 μmol/L (not including RRT patients), a prevalence of 12.2/1000. The authors concluded that the duration of lithium therapy was the only risk factor for developing CKD and ESRD. Long-term treatment was defined as a duration of ≥15 years on lithium. It was found that 16 (89 %) of the lithium-treated RTT patients had received lithium for more than 15 years. Similarly, lithium-treated patients who had plasma creatinine levels above 150 μmol/L were older than the general population of lithium-treated patients (average age 68 versus 56 years, respectively). The results of this retrospective study (Bendz et al. 2010) support a positive correlation between long-term lithium therapy and development of CKD (Aiff et al. 2014b).

Reduced GFR may sometimes occur at early stages after initiation of lithium therapy. For example, a prominent reduction in GFR was found among 53 patients who received lithium for only 4 months (Smigan et al. 1984). Lepkifker et al. (2004) studied 114 patients who had been taking lithium for 4 to 30 (mean 16.75) years. The control group included 94 untreated non-psychiatric subjects matched for sex and age (mean duration of follow-up 11.9 years). Ninety lithium-treated patients (79%) showed no increase in creatinine levels during a follow-up of up to 30 years and were similar to the control group. The other 24 patients had a progressive increase in creatinine levels during the follow-up period and were classified as having renal insufficiency (Lepkifker et al. 2004). The development of renal insufficiency was associated with episodes of lithium intoxication, other diseases, and/or drugs that affect renal function but not with the duration of lithium treatment in a majority of patients (Lepkifker et al. 2004).

The above reviewed evidence seems contradictory and difficult to summarize. Aiff et al. (2014a) suggested a potential solution to the contradictions: They found using their large Swedish registry that no patient treated since 1980 has developed ESRD in their sample and concluded that low-dose treatment apparently in use since then in Sweden does not cause ESRD. This is supported by the results of Aprhamian et al. (2014) who found no changes in kidney function after 2 years of low-dose lithium in elderly patients. However, Close et al. (2014) did not find this “time of treatment” effect in the UK in their large registry study. This “historical” approach does not explain the very early studies that found no effects of lithium in kidney biopsy. A general recommendation to use low-dose lithium, while consistent with the medical principle of *primum non nocere* and medical caution, does not help the clinician with difficult patients who respond fully only to high-dose maintenance. High-dose lithium has been reported to have increased efficacy in both prophylaxis and bipolar depression. Clearly, individual patient history of response and severity of episodes must be critical in the decision-making (Belmaker et al. 2012).

In summary, the evidence attests to a small but definite risk of lithium-induced reduction in GFR and progression to CKD. Some studies indicate that the risk for CKD and ESRD is low while others suggest that long-term lithium treatment increases the risk for chronic nephropathy to a clinically relevant degree.

### Case histories

Three of the authors (RHB, YB, YO) have maintained a bipolar clinic including lithium therapy for over 30 years servicing a defined catchment area of 300,000 people in southern Israel (Osher et al. 2010). We reviewed our charts for evidence of patients who had developed kidney disease:

1. ST (female, 65 years old). The patient was started on 1500 mg/day lithium at age 34 following hospitalization for acute mania. She was stable for 13 years. After the appearance of polyuria, the dose was reduced to 900 mg/day. Five years later, following a depression and reinstatement of 1500 mg/day, blood level rose to 1.8 mEq/L and creatinine levels reached 1.8 mg/dL. Nephrology consultation diagnosed mild renal failure, and the patient was switched gradually to valproate (800 mg/day); after which, no further progression of kidney damage was observed. Nine years after complete cessation of lithium, however, creatinine rose to 3.5 mg/dL and the patient began dialysis treatment 3 years ago. She is currently stable on valproate, awaiting a kidney transplant.
2. OE (male, 38 years old). The patient was first hospitalized at age 25 with a severe psychotic mania. Symptoms proved very difficult to control without high doses of lithium (up to 2550 mg/day) plus valproate and dopamine blockers (olanzapine or clozapine). Patient relapsed with psychotic symptoms any time blood lithium levels fell below 1.2 mEq/L. Creatinine levels rose from 1.1 to 1.4 mg/dL and blood urea nitrogen (BUN) from 27 to 43 mg/dL over a period of a few months. Lithium dose was lowered to 1500 mg/day, but the kidneys continued to deteriorate. Additional decreases of lithium dosage were postponed due to the objections of the patient and family. Recently, after the introduction of asenapine 30 mg/day in addition to olanzapine 10 mg and valproate 2500 mg/day, lithium has been reduced to 600 mg/day. Creatinine levels are currently 1.5 mg/dL, BUN = 53 mg/dL, and the patient is reasonably stable.
3. MBD (female, 77 years old). First hospitalized with severe depression at age 28, this patient began treatment with lithium (plus clomipramine) after a second hospitalization at age 50. She was stable for many years on lithium 900 mg/day, with blood levels of 0.5–0.6 mEq/L. Rheumatoid arthritis developed and was treated with azathioprine or gold; during the 6 years she received this treatment, lithium dosage was raised to 1200 mg/day and blood levels ranged from 0.6 to 1.2 mEq/L. The dosage was subsequently returned to 900 mg/day. Parkinson’s disease developed and was treated with rasagiline, carbidopa, and levodopa. When creatinine levels of 2.07–3.2 mg/dL and BUN of 66 mg/dL were observed, lithium was reduced to 150 mg/day. Creatinine and BUN levels began to improve. Shortly thereafter, at age 77, the patient left the area and was lost to follow-up.
4. JL (female, 60 years old). First hospitalized at age 32 due to a manic psychosis, this patient had an additional hospitalization less than a year later. Lithium was started at age 34, and the patient had full remissions (with four hospitalizations) for 17 years. When hospitalized again at age 51, lithium treatment was stopped (due to “lack of efficacy”) and treatment was switched to valproic acid plus perphenazine and afterwards continued as perphenazine alone. After 5 years of fragile stability and several mood episodes which did not require hospitalization, the patient was hospitalized again at age 56 and lithium was reinstated at a low dose (600 mg/day) plus 4 mg perphenazine. One year later, creatinine rose above 1.0 mg/dL. The patient has been affectively stable for the past 4 years, and creatinine is currently 1.5 mg/dL.
5. DL (female, 37 years old). While in her early 20s, this patient experienced major depression and attempted suicide. She was treated with valproate plus fluoxetine, which precipitated a manic episode and hospitalization. After 2 years of failed attempts at stabilization with valproate plus haloperidol or ziprasidone, including substantial weight gain, the patient was switched to lithium plus risperidone. A miscarriage and subsequent depression was treated with lithium 2100 mg/day plus perphenazine, with lithium blood levels reaching 1.5 mEq/L. The patient went through a successful pregnancy and delivery while being managed on lithium, but after 3 years, she developed nephrogenic diabetes insipidus. She was gradually switched over to lamotrigine (400 mg/day) plus perphenazine 8 mg/day and has been reasonably stable for 4 years, with creatinine levels of 1.0 mg/dL.
6. SD (male, 47 years old). The patient began treatment with carbamazepine plus haloperidol after a severe psychotic episode at age 25. After several additional hospitalizations in quick succession, lithium was added to the treatment, at first 1800–2100 mg/day then later reduced to 900 mg/day. Initial creatinine level was measured at 1.0 mg/dL but rose to 2.5–2.7 mg/dL after being on lithium for 10 years. Nephrogenic diabetes insipidus and renal failure developed during a 3-month period in which the patient was lost to follow-up. Lithium treatment was stopped; carbamazepine was continued. Patient was again lost to follow-up for 1 year, and at the end of which, he was hospitalized and diagnosed with end-stage renal failure (creatinine = 7.2 mg/dL). Treatment was changed to chlorpromazine, and the patient began renal dialysis and subsequently underwent a kidney transplant, which failed after 1 year. He is currently stable on chlorpromazine plus diazepam and is wait-listed for a second kidney transplant.
7. RP (male, 64 years old). Hypomanic episodes were experienced along with numerous hospitalizations for depression between the ages of 23 and 53. The patient was treated with a variety of medications and interventions not including lithium. Prior to hospitalization resulting from a suicide attempt at age 53, the patient had been treated with carbamazepine plus clonazepam. During the hospitalization, treatment was changed to lithium (1200 mg/d). The patient was stable for 8 years, but when creatinine level rose to 1.88 mg/dL and BUN to 42 mg/dL, lithium was reduced to 600 mg/day and then discontinued in favor of a combination of valproate and carbamazepine. He has been reasonably stable on this combination for approximately 3 years. Creatinine level has reduced slightly (currently 1.55 mg/dL) and BUN is currently 36 mg/dL.
8. SP (male, 66 years old). The patient’s first affective episode, at age 18, included a switch from depression to mania and a suicide attempt. He was treated with lithium and thioridazine for 29 years, with a full remission and only one additional hospitalization, until developing nephrogenic diabetes insipidus. Lithium was stopped and treatment switched to valproic acid plus topiramate and risperidone. The patient could not be stabilized, although multiple agents were tried, and experienced six hospitalizations during the following 5 years. Lithium was reinstated (900 mg/day) when the patient was 56 years old. One year later, creatinine was 1.59 mg/dL but rose quickly to 2.3 mg/dL within a few months. Lithium dose was reduced to between 600 and 750 mg/day, with blood levels of 0.9–1.1 mEq/L, and patient was stable with only one brief hospitalization over a period of 7 years. At that time, with creatinine at 2.06 mg/dL and lithium blood levels of 0.85 mEq/L although the dose had been reduced to 300 mg/day, it was decided again to stop lithium. The patient became severely manic within a few months in spite of treatment with relatively high doses of atypical antipsychotic medication, requiring hospitalization. Lithium was reinstated at doses of 300–600 mg/day, and the patient has remained reasonably stable for 3 years. Creatinine levels have continued to increase to 2.8 mg/dL and BUN 83 mg/dL, and the patient is currently being followed by a nephrologist while continuing treatment with lithium at low doses plus olanzapine.
9. SH (female, 66 years old). This patient, with a strong family history for bipolar disorder, experienced a psychotic postpartum depression at age 20 and began lithium prophylaxis following a successful course of electroconvulsive therapy. Lithium was continued with good remissions (though some recurrent affective episodes and hospitalizations). Hypothyroidism and parathyroid adenoma were treated by an endocrinologist without the need to stop lithium; the patient also had surgery for a cancerous growth in her breast. After 39 years of lithium treatment, the patient developed renal insufficiency with creatinine clearance of 50 ml/min, creatinine level = 1.6 mg/dL. She was successfully switched over to valproate (800 mg/day) approximately 5 years ago. She has been stable, with valproate levels in the 80s; creatinine is currently 2.0–2.2 mg/dL and BUN = 65 mg/dL.
10. LR (male, 54 years old). First hospitalized for an acute psychotic episode at age 17, this patient experienced multiple hospitalizations over a period of 7 years until lithium was added to his treatment. He remained essentially stable for 28 years while being treated with a combination of lithium 1500 mg/day plus chlorpromazine. Complications of treatment occurred over time, including hypertension and hyperthyroidism, and at age 52, he was diagnosed with chronic renal failure (creatinine = 1.62 mg/dL, BUN = 44 mg/dL). The patient was switched to treatment with valproate 2000 mg/d plus risperidone 3 mg/day and is currently stable with creatinine level of 1.75 mg/dL.
11. RB (male, 68 years old). Although known to have a “bad temper” and a positive family history for affective disorder, this patient was not diagnosed as having bipolar disorder until a first hospitalization for mania at age 50. He was stabilized on lithium 1500 mg/day plus low doses of various dopamine blockers. Lithium dosage was reduced to 600 mg/day when blood levels reached 1.4 mEq/L; creatinine clearance at the time was 70 mL/min, creatinine = 1.4 mg/dL, and BUN = 47 mg/dL. Stopping lithium was discussed, but due to a difficult bereavement, it was decided to continue treatment as before. The patient was stable after 1 year on lithium 300 mg/day plus risperidone 2 mg/day, with slight reduction in creatinine level (to 1.23 mg/dL) and BUN unchanged at 48 mg/dL.
12. YA (male, 66 years old). Although pronounced episodes of depression and hypomania were evident from the age of 33, with severe effects on his occupational and personal life, this patient was first diagnosed with bipolar disorder during his first hospitalization, at age 41 (for mania). He began treatment with 1200 mg/day lithium and was stable for 9 years. Due to inconsistent adherence, valproate was added at that point and lithium reduced to 900 mg/day. Lithium was reduced to 600 mg/day when creatinine level rose to 1.2 mg/dL (BUN = 34 mg/dL) and polyuria appeared. The patient became unstable when attempts were made to further lower lithium dose, until olanzapine (5 mg/day) was added to the treatment. One year later, the patient developed diabetes mellitus. Creatinine rose slightly (to 1.3 mg/dL, BUN = 34 mg/dL), and lithium was reduced to 300 and finally to 150 mg/day (the patient is extremely reluctant to stop lithium entirely). Although affectively stable, the patient has developed early-onset dementia, apparently due to cerebral aneurysm.
13. MO (male, 52 years old). Two years after a first hospitalization for psychotic mania at age 24, this patient with a positive family history of affective disorder began treatment with lithium. At age 43, he attempted suicide by ingesting 33 lithium tablets; creatinine level at that time was 1.4 mg/dL and BUN 34 mg/dL. Two years later, the patient again attempted suicide by ingestion of 55 lithium tablets. Lithium treatment was then terminated due to renal failure (creatinine = 1.8 mg/dL, BUN = 49 mg/dL), and the patient was switched over to clozapine. Creatinine and BUN levels continued to rise despite the cessation of lithium. In addition to chronic renal failure due to lithium poisoning, the patient also suffers from hyperthyroidism, hypercalcemia, dyslipidemia, and hypertension.
14. GG (male, 35 years old). Although apparently ill from his teens, this patient was first hospitalized and diagnosed with bipolar illness at age 26. He was eventually stabilized on lithium 1500 mg/day (blood levels 1.1–1.2 mEq/L) plus clozapine. Creatinine began to rise to 1.5 mg/dL, and lithium dosage was reduced to 900 mg/day and then stopped gradually over a period of 6 months. The patient is currently stable on clozapine, and creatinine is currently 1.3 mg/dL and BUN 24 mg/dL.
15. VV (female, 78 years old). This patient, with a strong family history of bipolar disorder, was first diagnosed (with mania) at age 58. For the following 17 years, she was treated with lithium (750–900 mg/day; blood lithium levels approximately 0.75 mEq/L). At age 75, she developed chronic renal failure, with creatinine = 1.4 mg/dL and BUN = 44 mg/dL. She continues to be treated with lithium at low doses (300–450 mg/day), with blood lithium levels 0.8–1.0 mg/day, and is affectively stable. Creatinine is currently 1.6 mg/dL and BUN = 62 mg/dL.

In summary, out of approximately 180 active patients currently in our affective disorders clinic, we have 2 patients on dialysis and another 13 with identified renal disorder. Over 90% of our patients have been exposed to lithium therapy at some point in time, although only roughly 60% are currently being treated with lithium, either as monotherapy or in combination with other medications. This translates to a very rough renal dysfunction prevalence of 7–8 %, similar to that reported by Close et al. (2014). However, the analysis of Close et al. (2014) suggests that one half to two thirds of this prevalence is caused in reality by factors other than lithium (Werneke and Ott 2014).

## Conclusions

Since chronic kidney disease exists in the population with many etiologies and its incidence increases with age, it was denied for many years that lithium is a specific cause of chronic kidney disease. The accumulated epidemiologic, biologic, and clinical evidence today weighs in favor of the concept that lithium does increase the risk for chronic kidney disease. The course of this effect is not clear. Some patients have slow increases in creatinine late in life and can be maintained on lithium on lower doses while other patients have severe increases in creatinine with decreases in kidney function that are progressive even after lithium is discontinued. The atypical antipsychotics that have been promoted as safe alternatives to lithium in the treatment of bipolar disorder have been revealed in many cases to have short-term effects on glucose and lipids and long-term risks of diabetes, side effects no less serious than the chronic kidney disease associated with lithium. On the other hand, some clinicians find lithium treatment uniquely useful and effective and deny or ignore the risk of chronic kidney disease. Clearly, there is no free lunch in pharmacological treatment of medical disorders. Short-term and long-term side effects are correlated although not entirely. Just as weight gain and hyperlipidemia predict the risk of diabetes after long-term quetiapine treatment but not so well as to eliminate the risk in a quetiapine patient without weight gain, thus, nephrogenic diabetes insipidus and episodes of lithium intoxication may partially predict long-term development of chronic kidney disease, but the absence of these risk factors does not eliminate the risk. In general, the use of higher lithium doses in the past may be responsible for much of the lithium toxicity reported even now in epidemiological studies. It is best to use the lowest blood level effective in each particular patient (American National Kidney Foundation 2002).

For some patients with bipolar disorder who have lithium-responsive bipolar patients in the family, lithium is an absolute first choice of treatment. Other patients will receive lithium because of the side effects with anticonvulsants or atypical antipsychotics or lack of efficacy with those compounds. Chronic kidney disease may occur in such patients and its risks minimized by appropriate clinical monitoring. There is no specific biopsy finding that at present would allow us to definitively ascribe those cases that do develop chronic kidney disease to the lithium treatment rather than to other factors that cause chronic kidney disease in the general population.

A critical question (Werneke et al. 2010) is when to stop lithium in a patient who develops impairment in kidney function. Such a decision should not be based exclusively on whether a patient has a slowly increasing creatinine. Two key factors may help guide the decision to stop lithium: 1) Whether the past treatment history or family histories suggest that it is possible to switch the patient to an alternative therapy without significantly increasing the risk of relapse or suicide. If the patient is a good responder to lithium and cessation of lithium will drastically increase the risk of affective deterioration, then the therapeutic efficacy of lithium may outweigh the risk of nephrotoxicity. If another therapy known to be effective in this patient is available, lithium cessation should be considered. However, it should be noted that other mood stabilizer medications also have serious side effects and are not necessarily safer than lithium. 2) Whether cessation of lithium will avoid further deterioration in kidney function. If assessment of kidney function suggests reversible damage and possibility of stabilization or improvement, then it is reasonable to consider stopping lithium. In most patients it is impossible to predict whether discontinuation of lithium will result in stabilization of kidney function. If the renal impairment is irreversible, discontinuation of lithium will not benefit the kidneys but may increase the risk of relapse and thus should be avoided. NDI, even when severe, or increase in creatinine levels or decrease in creatinine clearance, is almost never an indication for abrupt lithium discontinuation. There is no medical emergency and discontinuation if decided upon can be very gradual with overlap of alternative therapy.

New mood stabilizers that are alternatives to the use of lithium first came into the field from the anticonvulsant world where valproate, carbamazepine, and lamotrigine all seem to have mood-stabilizing properties. More recently, the atypical antipsychotics including olanzapine, quetiapine, aripiprizole, and lurasidone (Belmaker 2014) have all been shown to prevent manias and depressions. None of these agents show overall superiority to lithium although lithium is not overall superior to these alternatives. Each alternative presents its own unique and serious side effect profile. Despite the fact that lithium is an unpatented compound and that newer patented compounds have been marketed vigorously, psychiatrists in the developed world seem to continue to prescribe lithium to a significant fraction of bipolar patients. This may be because some difficult-to-define subgroup of bipolar patients is particularly responsive to lithium or because the side effect profile of lithium is more acceptable to some patients. The mechanism of action of lithium is unique among the mood stabilizers since it is neither an anticonvulsant nor a dopamine D2 blocker. Its mechanism of action is not known but it has major effects on inositol monophosphatase and glycogen synthase kinase (Toker et al. 2014).
